# Gas6 Inhibits Toll-Like Receptor-Mediated Inflammatory Pathways in Mouse Microglia *via* Axl and Mer

**DOI:** 10.3389/fncel.2020.576650

**Published:** 2020-10-09

**Authors:** Shannon E. Gilchrist, Salman Goudarzi, Sassan Hafizi

**Affiliations:** School of Pharmacy and Biomedical Sciences, University of Portsmouth, Portsmouth, United Kingdom

**Keywords:** microglia, Gas6, TAM receptors, toll-like receptors, LPS, inflammation, TNF-α, primary cell culture

## Abstract

**Background**: Microglia are well known key regulators of neuroinflammation which feature in multiple neurodegenerative disorders. These cells survey the CNS and, under inflammatory conditions, become “activated” through stimulation of toll-like receptors (TLRs), resulting in changes in morphology and production and release of cytokines. In the present study, we examined the roles of the related TAM receptors, Mer and Axl, and of their ligand, Gas6, in the regulation of microglial pro-inflammatory TNF-α production and microglial morphology.

**Methods**: Primary cultures of murine microglia of wild-type (WT), Mer^−/−^ and Axl^−/−^ backgrounds were stimulated by the TLR4 agonist, lipopolysaccharide (LPS) with or without pre-treatment with Gas6. Gene expression of TNF-α, Mer, and Axl was examined using reverse transcription-quantitative polymerase chain reaction (RT-qPCR), and enzyme-linked immunosorbent assay (ELISA) was used to measure TNF-α release from microglia. Immunofluorescence staining of β-actin and the microglial marker Iba1 was performed to reveal microglial morphological changes, with cellular characteristics (area, perimeter, Feret’s diameter, minimum Feret, roundness, and aspect ratio) being quantified using *ImageJ* software.

**Results**: Under basal conditions, TNF-α gene expression was significantly lower in Axl^−/−^ microglia compared to WT cells. However, all microglial cultures robustly responded to LPS stimulation with the upregulation of TNF-α expression to similar degrees. Furthermore, Mer receptor expression was less responsive to LPS stimulation when in Axl knockout cells. The presence of Gas6 consistently inhibited the LPS-induced upregulation of TNF-α in WT, Mer^−/−^ and Axl^−/−^ microglia. Moreover, Gas6 also inhibited LPS-induced changes in the microglial area, perimeter length, and cell roundness in wild-type cells.

**Conclusion**: Gas6 can negatively regulate the microglial pro-inflammatory response to LPS as well as *via* stimulation of other TLRs, acting through either of the TAM receptors, Axl and Mer. This finding indicates an interaction between TLR and TAM receptor signaling pathways and reveals an anti-inflammatory role for the TAM ligand, Gas6, which could have therapeutic potential.

## Background

Neuroinflammation is a common feature of disorders such as multiple sclerosis or Alzheimer’s disease, among others, occurring at various stages of disease progression (Weggen et al., 2001; Frischer et al., 2009; Heneka et al., 2014; Dendrou et al., 2015). Microglia, the principal immune cells of the CNS, are responsible for surveying the CNS environment in search of damage (Nimmerjahn et al., 2005; Salter and Stevens, 2017). Upon activation, microglia can detect a variety of stimuli through detecting damage- or pathogen-associated molecular patterns (DAMPs or PAMPs, respectively) and respond by creating an inflammatory milieu through the release of cytokines such as TNF-α and IL-1β (Lively and Schlichter, 2018; Subedi et al., 2019). In inflamed tissue, microglia can control the release of pro-inflammatory cytokines, switching to an anti-inflammatory response to promote healing (Heneka et al., 2014). Moreover, microglia undergo morphological changes upon inflammatory stimulation. The transition from their surveillant state to a more “classically” activated phenotype has been well characterized with cells switching from a ramified, extended morphology to a more rounded, amoeboid structure (Djukic et al., 2006; Tam and Ma, 2014; Michell-Robinson et al., 2015; Arcuri et al., 2017). It is this molecular and phenotypic regulation of microglia that may become inefficient during neurodegeneration as chronic inflammation takes hold (Song and Colonna, 2018).

Toll-like receptors (TLRs) are a family of pattern recognition receptors (PRRs), present on the surface of microglia (Bsibsi et al., 2002; Olson and Miller, 2004; Frederiksen et al., 2019). TLR4, for which the gram-negative bacterial cell wall component lipopolysaccharide (LPS) is a strong agonist, is well known for its role in inflammation, providing many opportunities for therapeutics (Jack et al., 2005; Lu et al., 2008; Roy et al., 2016). The TAM (Tyro3, Axl, Mer) subfamily of receptor tyrosine kinases (RTKs) are known to play an important role in the resolution of inflammation, both within and outside the CNS (Lemke and Rothlin, 2008; Shafit-Zagardo et al., 2018; Lee and Chun, 2019). For example, Mer^−/−^ mice treated with LPS show an exacerbated sickness behavior (Camenisch et al., 1999). Furthermore, TAM receptors, along with their ligand Gas6, have been implicated in the negative regulation of pro-inflammatory cytokine release from LPS-stimulated cells (Binder et al., 2008; Alciato et al., 2010).

Within the CNS, TAM receptor expression is highly variable concerning both age and cell type. Microglia have a strong expression of both Axl and Mer receptors but a negligible expression of Tyro3, especially neonatally (Prieto et al., 2000; Shafit-Zagardo et al., 2018; Goudarzi et al., 2020). Although Axl and Mer, along with Gas6, are known to play important roles in the macrophage/microglial inflammatory response, little has been confirmed regarding the specific roles that each receptor plays in the resolution of the response, nor their actions on microglial morphological responses. Therefore, this study aimed to investigate the specific roles of the TAM receptors, Mer and Axl, in regulating TNF-α expression and release in primary mouse microglia as well as to explore the impact of Gas6 on microglial morphological changes under pro-inflammatory conditions. Here, we have used single receptor knockout microglia to study both cytokine release in response to pro-inflammatory stimulation and morphological effects, with and without the influence of ligand stimulation by Gas6. We discovered that both Mer and Axl play important roles in inflammatory resolution and that Gas6 can inhibit a broad range of TLR-mediated inflammatory induction of TNF-α as well as associated morphological changes.

## Materials and Methods

### Primary Mouse Microglial Cell Cultures

All experimental procedures were performed following the Animals (Scientific Procedures) Act, 1986 under a UK Home Office project license (license number PC2238199) with approval from the institutional ethics committee (AWERB).

Primary microglial cells were derived according to Mecha et al. (2011). In brief, cells were isolated from neonatal (P1–3) wild-type (WT; C57/BL6), Mer mutant (Mer^−/−^; Jackson Laboratories, Bar Harbor, ME, USA), and Axl mutant (Axl^−/−^; Jackson Laboratories) mouse brains and cultured in Dulbecco’s Modified Eagle Medium (DMEM; Thermo Fisher Scientific, Loughborough, UK) supplemented with 10% fetal bovine serum (FBS; Lonza, Slough, UK), 10% horse serum (HS; Gibco Invitrogen, Paisley, UK) and 1% penicillin/streptomycin (P/S; Thermo Fisher Scientific) at 37°C for 10–14 days. Cultures were then shaken orbitally at 260 rpm for 3 h to detach the microglia, which were seeded onto poly-D-lysine coated 24-well plates at a cell density of 3 × 10^5^ cells/well for protein/RNA extraction protocols or onto coverslips (circular, 16 mm diameter; Thermo Fisher Scientific) at 4 × 10^4^ cell density for immunofluorescence staining.

Cells were allowed to adhere for 1–2 days before the medium was changed to DMEM containing 1% FBS and 1% P/S into which treatments were added. Pro-inflammatory stimulation was induced by adding LPS (10 ng/ml; Sigma–Aldrich, Gillingham, UK) to cells. Other cell treatments included the TAM receptor ligand, Gas6 (1.6 μg/ml; produced in house as detailed in Stenhoff et al., 2004).

### RNA Extraction, Reverse Transcription, and qPCR

After experimental treatments, media was removed from wells, and cells were lysed for total RNA extraction and purification using the RNeasy Mini Kit (Qiagen, Hilden, Germany) according to the manufacturer’s instructions. RNA concentration and purity were measured using a spectrophotometer (ND-1000; NanoDrop Technologies, Wilmington, DE, USA). Equal amounts of total RNA were reverse transcribed (RT) into cDNA (High Capacity cDNA Reverse Transcription Kit, Applied Biosystems, Foster City, CA, USA) which was used to perform quantitative polymerase chain reaction (qPCR) using gene-specific primer/fluorescent hydrolysis probe sets (Integrated DNA Technologies, IDT, Leuven, Belgium). Gene expression was normalized to *Gapdh* (Thermo Fisher Scientific) as a reference gene in each sample; relative gene expression was calculated using 2^−ΔCt^ and fold change in expression was calculated using 2^−ΔΔ^ for different experiments, as previously described (Schmittgen and Livak, 2008).

### Enzyme-Linked Immunosorbent Assay (ELISA)

Legend Max™ Mouse TNF-α Enzyme-Linked Immunosorbent Assay (ELISA) Kit (BioLegend, San Diego, CA, USA) was used following the manufacturer’s instructions to measure protein levels in cell-conditioned media. In brief, the medium was added to a 96-well plate pre-coated with hamster monoclonal anti-mouse TNF-α capture antibody. After incubation at room temperature for 2 h, samples were washed and the detection antibody was added for a further hour. After further washing, avidin-horseradish peroxidase was used for detection and optical density was measured at 450 nm and 570 nm (Multiskan GO; Thermo Fisher Scientific).

### Immunofluorescence Staining

After treatments, media was removed from coverslips by aspiration, and cells were washed twice with PBS, then fixed by incubation with 4% paraformaldehyde (PFA; Sigma–Aldrich) in PBS for 10 min at room temperature. Cells were then washed in PBS (3 × 5 min) before permeabilization with 0.3% Triton X-100 at room temperature for 5 min. Coverslips were washed again (3 × 5 min) before further permeabilization using 100% methanol at −20°C for 10 min. After a final wash, cells were blocked in 5% HS for 1 h.

For staining, coverslips were incubated in primary antibodies made in 1% bovine serum albumin (BSA) solution to appropriate dilutions at 4°C overnight. Primary antibodies were removed, coverslips were washed and secondary antibodies in 1% BSA were added for 1 h at room temperature. Coverslips underwent a final wash in PBS before being mounted onto clear glass microscopy slides using PermaFluor Aqueous Mounting Medium (Thermo Fisher Scientific). Primary antibodies used were rabbit anti-Iba1 (1:300; Fujifilm Wako Pure Chemical Corporation, Japan) and mouse anti-β-actin [1:2,500; Cell Signaling Technologies (CST), London, UK]. Secondary antibodies used were anti-rabbit AlexaFluor 488 (1:1,000; Invitrogen, UK), anti-mouse Cy3 (1:1,000; Invitrogen), and the stain DAPI was used at 1:400 (Invitrogen Molecular Probes). Images were taken on a laser scanning confocal microscope (Zeiss LSM 710, Cambridge, UK).

### Image Processing

The program RBS *ImageJ* was used for all image processing. Firstly, all images were scaled to micrometers and converted to black and white (B&W) images using “Image… Colour Threshold.” Cellular characteristics were then measured using “Analyze…Analyze Particles” where numbered outlines were created for each cell over 100 μm^2^ that did not cross the image threshold (Supplementary Figure 1) and parameters were automatically measured. Parameters used were area, perimeter, Feret’s diameter, minimum Feret, roundness, and aspect ratio as described in Zanier et al. (2015). A subtype of giant multinucleated phagocytic cells (Fendrick et al., 2007; Hornik et al., 2014) were excluded from data analysis due to the limited number observed in images. Furthermore, extreme data outliers and any cells in contact with others were removed from the analysis. In total, over 300 cells were analyzed for each treatment group.

### Statistical Analysis

Statistical analysis was performed using Prism 8 (GraphPad Software, San Diego, CA, USA). The normal distribution of experimental data was determined using the Shapiro–Wilk test. Statistical tests used on normally distributed data were one-way analysis of variance (ANOVA) or paired Student’s *t*-tests, whereas non-parametric tests used were Kruskal–Wallis, Friedman’s, or Wilcoxon signed-ranked tests. A *p* ≤ 0.05 was considered as statistically significant. Data analyzed were from a minimum of four independent experiments on separate primary cultures.

## Results

### Axl Knockout Microglia Show Reduced Baseline TNF-α Expression, but Wild-Type and TAM Knockout Cells Respond Equally to LPS

**Figure 1:**
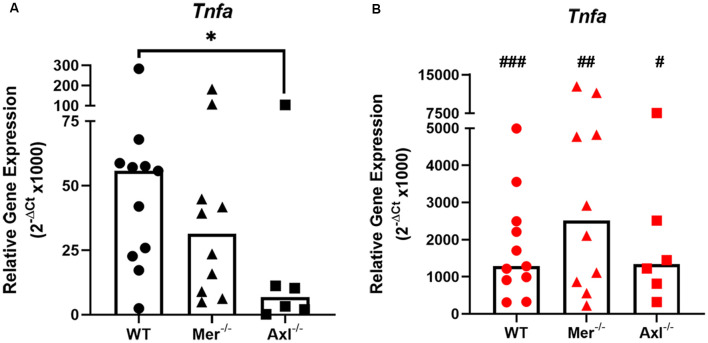
Reduced baseline but not an inflammatory-stimulated expression of TNF-α in Mer and Axl deficient microglia. Reverse transcription-quantitative polymerase chain reaction (RT-qPCR) was used to determine the relative gene expression (2^−ΔCt^) of TNF-α in wild-type (WT), Mer^−/−^ or Axl^−/−^ microglia when **(A)** unstimulated or **(B)** stimulated with lipopolysaccharide (LPS; 10 ng/ml) for 8 h. Gapdh was used as a reference gene. Data shown are individual data points with a median bar for *n* ≥ 6 independent experimental repeats. Statistical significance was determined using Mann-Whitney test comparing Mer^−/−^ or Axl^−/−^ microglia against WT (*) or by using Wilcoxon signed-rank tests to compare pairwise differences between basal and lipopolysaccharide (LPS) values (#); *p < 0.05; ^#^*p* < 0.05; ^##^*p* < 0.01; ^###^*p* < 0.005.

To discover the role of Mer and Axl receptors in intrinsic TNF-α expression in primary microglia, reverse transcription-quantitative polymerase chain reaction (RT-qPCR) was used to measure TNF-α gene expression in non-stimulated microglia of WT, Mer^−/−^ and Axl^−/−^ backgrounds. Under basal conditions, Axl^−/−^ microglia displayed significantly lower TNF-α gene expression when compared with WT microglia, whereas Mer^−/−^ cells displayed no such difference (Figure 1A). LPS (10 ng/ml) addition to WT microglia for 8 h caused a massive upregulation of TNF-α gene expression through TLR4 activation (Roy et al., 2016), which also occurred to the same degree in both Mer^−/−^ and Axl^−/−^ microglia, all differing significantly from basal values (Figure 1B). We also observed this effect with a separate commercial LPS preparation (Supplementary Figure 2), which furthermore showed the TNF-α-inducing effect occurred mainly *via* distinct TLRs and not all equally.

### Pro-inflammatory Stimulation by LPS Downregulates Mer but Upregulates Axl Expression in Microglia, With Axl Proving Necessary for This Response

**Figure 2:**
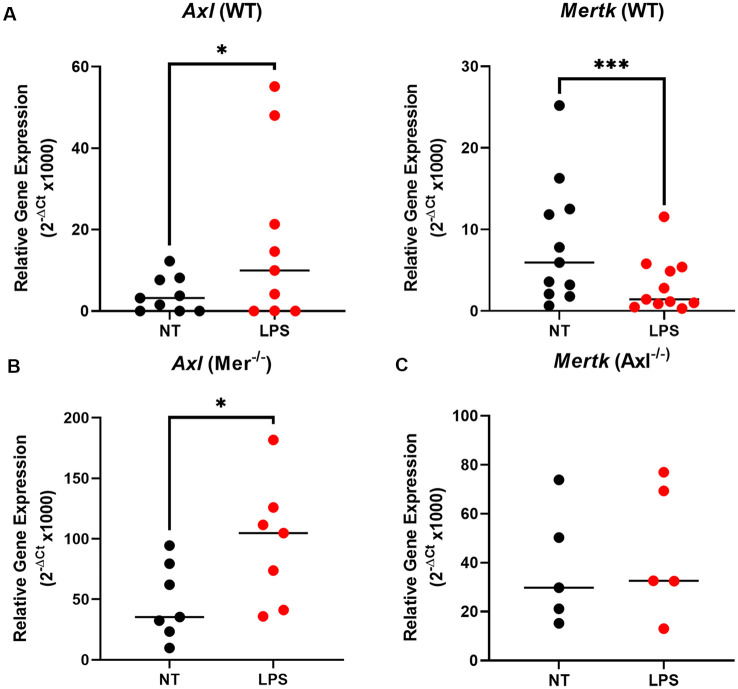
LPS stimulation alters TAM receptor expression in opposite directions, with Axl regulating the Mer response. After 8 h LPS (10 ng/ml) stimulation or not, RT-qPCR was used to determine the relative gene expression (2^−ΔCt^) of **(A)** Axl and Mertk in WT microglia, **(B)** Axl in Mer^−/−^ microglia, and **(C)** Mertk in Axl^−/−^ microglia. Gene expression was normalized to Gapdh. Data shown are individual data points with the median line for *n* ≥ 5 independent experimental repeats. Statistical significance was determined using Wilcoxon signed-rank test; *p < 0.05; ***p < 0.001.

To understand the underlying alterations in TAM receptors in response to LPS, we measured gene expression of Mer and Axl receptors first in WT and then Mer^−/−^ and Axl^−/−^ microglia. In WT microglia, stimulation with LPS (10 ng/ml) for 8 h caused a significant upregulation of Axl expression concomitant with a significant downregulation of Mer (Figure 2A). Interestingly, the LPS-induced upregulation of Axl was also observed in Mer^−/−^ microglia (Figure 2B) whilst, in contrast, LPS did not alter Mer expression in Axl ^−/−^ cells (Figure 2C). This response indicates a potential regulatory role of Axl for the expression of Mer in microglia. Furthermore, under baseline, non-LPS-stimulated conditions, single knockout of either Mer or Axl receptor caused a significant increase in the other remaining TAM receptor (Supplementary Figure 3; *p* = 0.0032, Mer expression for WT vs. Axl^−/−^ baseline; *p* = 0.0003, Axl expression for WT vs. Mer^−/−^ baseline). This suggests a compensation between the TAM receptors when one is non-functional.

### Gas6 Inhibits LPS-Induced TNF-α Upregulation in Both Wild-Type and Knockout Microglia

**Figure 3:**
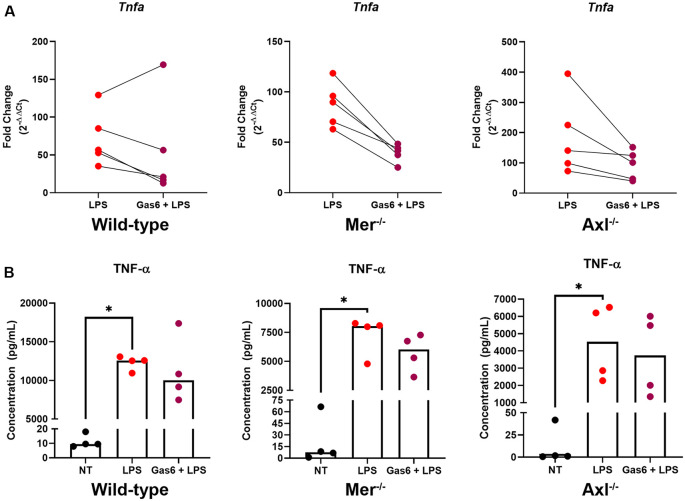
Gas6 inhibits TNF-α induction by LPS in WT and single TAM receptor knockout microglia. **(A)** RT-qPCR was used to determine the fold change (2^−ΔΔCt^) in TNF-α gene expression in WT, Mer^−/−^ or Axl^−/−^ microglia following 8 h of LPS (10 ng/ml) stimulation with or without 1 h pre-treatment with Gas6 (1.6 μg/ml); fold-change in expression for each experimental repeat (*n* = 5) is displayed. **(B)** Enzyme-Linked Immunosorbent Assay(ELISA) measurement of TNF-α protein in culture media under the same experimental conditions as in panel **(A)**, with individual data points from each separate culture and median bar displayed for *n* = 4 independent experimental repeats. Statistical tests used were **(A)** Wilcoxon signed-rank tests or **(B)** Friedman’s tests; *p < 0.05.

To further distinguish the roles of Mer and Axl receptors, we primed cells with their ligand, Gas6, before stimulating cells with LPS and measuring TNF-α expression. Initially, we confirmed the ability for LPS to almost completely suppress endogenous Gas6 expression in WT microglia (Supplementary Figure 4). This would mean that all experiments under LPS inflammatory conditions using exogenous Gas6 to activate Mer and Axl would be exclusively through exogenous and not endogenous Gas6. Under conditions of LPS stimulation, the presence of Gas6 throughout the 8-h incubation period (including 1 h preceding it) attenuated the upregulation of TNF-α gene expression in both WT and single TAM receptor knockout microglia (Figure 3A). This response occurred in a pairwise trend with the majority of samples showing a substantial down-regulation of gene expression (*p* = 0.4375, WT LPS vs. LPS + Gas6 vs; *p* = 0.0625, Mer^−/−^ LPS vs. LPS + Gas6; *p* = 0.0625, Axl^−/−^ LPS vs. LPS + Gas6). LPS also strongly induced the upregulation of TNF-α at the protein level as measured by ELISA analysis of TNF-α released from cells into the medium (Figure 3B). However, the Gas6 inhibitory effects observed by qPCR were not significant at the protein level. Also, both Axl^−/−^ and Mer^−/−^ cells showed significantly lower levels of LPS-induced TNF-α protein release than WT cells (*p* = 0.0286, LPS WT vs. LPS Axl^−/−^; *p* = 0.0286, LPS WT vs. LPS Mer^−/−^). The interference of Gas6 in signaling pathways regulating TNF-α expression was not unique to TLR4 activation, as we also observed the same inhibitory effect of Gas6 downstream of other TLR agonists (Supplementary Figure 2).

### Gas6 Limits Changes in Morphological Characteristics in Microglia in Response to LPS Stimulation

**Figure 4:**
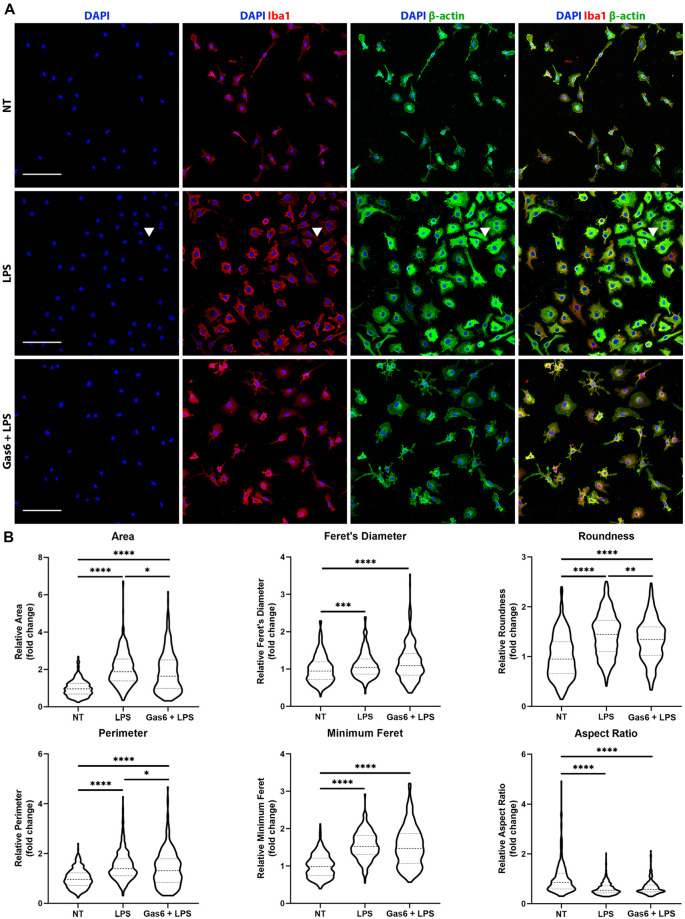
Gas6 counteracts LPS-induced changes in microglial morphology.** (A)** Representative images of primary microglial cultures treated with LPS (10 ng/ml for 19 h) with or without 1 h pre-treatment with Gas6 (1.6 μg/ml). Cells were stained with DAPI, Iba1, and β-actin and imaged under fluorescence confocal microscopy. Scale bar −100 μm. Arrowheads indicate a multinucleated cell. **(B)** Quantification of morphological characteristics (area, Feret’s diameter, roundness, perimeter, minimum Feret, and aspect ratio) for *n* = 4 individual experimental repeats (*n* > 300 cells per treatment group). Data normalized to the control no treatment (NT) group is displayed as fold change on violin plots showing median and upper/lower quartiles. Statistical tests used were one-way analysis of variance (ANOVA) with Tukey *post hoc* analysis; *p < 0.05; **p < 0.01; ***p < 0.001; ****p < 0.0001.

LPS is known to activate microglia to an M1 phenotype, resulting in them becoming more amoeboid in morphology (Tam and Ma, 2014). Here, we confirmed the LPS-induced transition to a more rounded morphology and investigated whether the changes in TNF-α expression observed with Gas6 pre-treatment also translated into morphological changes. WT microglia were seeded onto glass coverslips and treated with LPS (10 ng/ml), with or without 1 h Gas6 (1.6 μg/ml) pre-treatment, for 19 h (Figure 4). DAPI was used for nuclear staining, Iba1 to confirm the purity of microglial cultures, and β-actin to visualize the cytoskeleton and morphology of cells. Data were normalized for each experimental repeat to account for variation among cultures (original data and all images in Supplementary Figure 5). LPS stimulation of microglia had a profound effect on morphology, measured through several parameters (Figure 4B). Moreover, Gas6 pre-treatment of LPS-stimulated microglia significantly reverted the altered morphological parameters area, perimeter, and roundness towards the non-stimulated state, with cells displaying a less pronounced amoeboid shape.

## Discussion

In the present study, we have shown that the potent TLR4 agonist, LPS, strongly induced expression of the pro-inflammatory cytokine TNF-α in mouse microglia, in both WT cells and those deficient in either the Mer or Axl receptor. The absence of a single TAM receptor did not influence the ability of microglia to respond to this major pro-inflammatory stimulus, although basal TNF-α expression was reduced in Axl^−/−^ microglia. Interestingly, baseline expression of Mer or Axl was significantly higher in cells in which the other receptor was absent as compared to WT cells, suggesting a compensatory effect. Additionally, we observed that the TAM receptor ligand, Gas6, had an inhibitory effect on LPS-induced microglial TNF-α expression and release, independently of Mer or Axl receptor knockout. Finally, we observed significant alteration of microglial morphology in WT microglia in response to LPS which was also counteracted by Gas6.

The TAM receptor system is well established in immune processes, evident from the increased susceptibility of Mer^−/−^ mice to LPS-induced inflammation (Camenisch et al., 1999) and TAM receptor triple knockout mice displaying autoimmune characteristics (Lu and Lemke, 2001). The effects of TAM receptors on pro-inflammatory responses are often identified through alterations in cytokine expression or changes to intracellular signaling pathways (Rothlin et al., 2007; Ji et al., 2013; Zheng et al., 2015). In keeping with our observations, other studies have also reported the ability of individual TAM receptors to work in concert when regulating the cell’s response to stimuli. For example, Axl and Mer have been shown to work together for optimal clearance of apoptotic cell bodies by microglia (Fourgeaud et al., 2016), and phagocytosis by bone marrow-derived macrophages is dependent on both Mer and Axl kinase activity (Zagórska et al., 2014). Also, recent evidence supports the independent roles of Mer and Axl, whilst working together to suppress inflammatory responses (Zagórska et al., 2020). Furthermore, opposite responses of Mer and Axl to inflammatory conditions have been reported in human myeloid cells (Healy et al., 2016), with the upregulation of Axl being associated with a pro-inflammatory response (Gao et al., 2019).

The present study in microglia also concurs with previous reports of the negative regulatory role of Gas6 in inflammatory signaling in other cells. For example, Gas6 has previously been shown to have a suppressive effect on TLR-mediated pro-inflammatory cytokine production in cardiomyocytes (Grommes et al., 2008), microglial cell lines (Li et al., 2019), mouse macrophages (Deng et al., 2011) and primary murine microglia (Binder et al., 2008). There is growing evidence linking alleviation of inflammation to morphological alterations in microglia which are associated with pro-inflammatory signaling responses and cytokine expression (Zhang et al., 2014, 2019; Kalakh and Mouihate, 2017; Honjoh et al., 2019). This study used WT microglia to observe the potential modulatory effects of Gas6 on microglial morphological characteristics. We did not analyze morphology in TAM single knockout cells as their TNF-α release profiles under LPS conditions were similar to wild-type responses, as were the Gas6 effects on these; therefore, the morphological responses would be expected to be the same.

When measuring TNF-α protein release, the data concurred with the gene expression analysis in some areas but not others. For example, there were no significant differences between the LPS-induced TNF-α gene expression between the three groups of cell cultures, whereas under LPS stimulation, Mer^−/−^ and Axl^−/−^ cells showed lower TNF-α protein release than WT cells. This could be due to a maximal activation by LPS of the *Tnfa* gene promoter that is too strong to be affected by the lack of a single TAM receptor. In contrast, TNF-α protein release occurs at the end of a multi-level pathway to expression and therefore could be more sensitive to the absence of a TAM receptor through post-transcriptional mechanisms such as protein modification, stability, or trafficking (Vogel and Marcotte, 2012). Also, the lack of difference between baseline TNF-α protein release by WT and Mer^−/−^/Axl^−/−^ cells could be due to the poorer sensitivity of ELISAs for the detection of low levels of cytokine present in the supernatant, not taking into account cytokines that had been consumed by cells (Amsen et al., 2009). Also, we observed that active stimulation of either the TAM receptor by Gas6 is capable of suppressing the LPS effect at the gene level but not significantly at the protein level. In this case, constant ligand stimulation of TAM signaling pathways appear to have sufficient inhibitory efficacy to interfere with TNF-α gene promoter activation, whereas not sufficiently to affect levels of cytokine accumulated in the medium after a time period. In addition, it was noteworthy that under LPS stimulation conditions, and contrary to resting cells, knockout of a single TAM receptor did not decrease expression of the other. Therefore, single TAM levels were maintained at least at the unstimulated levels and these cells would be responsive to Gas6 stimulation, which can explain why Gas6 was also able to inhibit TNF-α expression in LPS-stimulated Axl^−/−^ cells, as their Mer expression remains robust.

There are other aspects of the TAM system, receptor interactions, and their involvement in inflammation touched on in our work. For example, the distinct responses of Mer and Axl to inflammatory stimuli suggest potential heterodimerization, previously proposed when protein S, a Mer and Tyro3 ligand, was able to stimulate an Axl response (Ray et al., 2017). It is important to know how the different components interact before a full understanding of the TAM receptor system is possible. It is also worth noting that this study focussed solely on Gas6, a ligand for all three TAM receptors, and not protein S (Tsou et al., 2014; Al Kafri and Hafizi, 2019) which has also been purported to have anti-inflammatory functions (Carrera-Silva et al., 2013; Barth et al., 2018). Furthermore, we have shown here that Gas6, *via* the TAM receptors Axl and Mer, negatively regulates the inflammatory response downstream of multiple TLRs, and other studies are continuing to elucidate these interactions (Wu et al., 2018; Herrera-Rivero et al., 2019; Zahoor et al., 2020). It is vital to probe further to fully clarify how the TAM system is involved in pro-inflammatory cytokine mediation and the underlying signaling pathways involved. This understanding could provide a fresh avenue for the treatment of many debilitating neuroinflammatory disorders.

In this study, we have used the pro-inflammatory cytokine TNF-α as a prominent biomarker to investigate proinflammatory responses (Muhammad, 2019). It is the case however that microglial responses to inflammation involve the complex release and functional interplay of many different cytokines (Lively and Schlichter, 2018). However, our aim here was to gain a clear view of the role of Gas6 in regulating a major pro-inflammatory mechanism, and with future work to be directed at investigating the key signaling pathways and inflammatory markers involved in the TLR response. Also in this study, analysis of TAM receptor protein expression may have supplemented the more sensitive gene expression data; however, the lack of highly sensitive anti-mouse TAM receptor antibodies coupled with the low levels of protein present in the samples precluded a reliable analysis to be done on all the cultures as they were using qPCR. Furthermore, blockade of Gas6 may have been a useful tool to allow us to confirm any ligand-dependent roles of the TAM receptors that occur under basal conditions in microglial cells. However, currently there are no specific Gas6 ligand traps available, and therefore it was not possible to completely negate endogenous Gas6 effects during our experiments. Nevertheless, our data show the clear modulatory role of Gas6 under pro-inflammatory conditions, which are the main findings of this study.

To conclude, we have shown that Gas6, *via* both TAM receptors Mer and Axl, has a significant negative regulatory influence on the pro-inflammatory response in primary mouse microglia, as observed through TNF-α expression and microglial morphology, and downstream of multiple TLRs. Furthermore, pro-inflammatory conditions alter TAM receptor expression in opposite directions and Axl appears to play a role in the regulation of Mer expression. These findings have expanded the current knowledge on the anti-inflammatory capabilities of Gas6 and TAM receptors in microglia. This new knowledge is of relevance to a greater understanding of the pathophysiology of several neurodegenerative diseases as well as potential novel therapeutic avenues.

## Data Availability Statement

The datasets used and/or analyzed during the current study are available from the corresponding author on reasonable request.

## Ethics Statement

The animal study was reviewed and approved by UK Home Office project licence (number PC2238199); and University of Portsmouth ethics committee (AWERB).

## Author Contributions

SH, SG, and SEG conceived and designed the experiments. SEG conducted the experiments, analyzed the results, and drafted the manuscript. SH contributed to data interpretation and manuscript writing. All authors contributed to the article and approved the submitted version.

## Conflict of Interest

The authors declare that the research was conducted in the absence of any commercial or financial relationships that could be construed as a potential conflict of interest.

## Abbreviations

ANOVA: analysis of variance
B&W: black and white
BSA: bovine serum albumin
CST: Cell Signaling Technologies
DAMPs: damage-associated molecular patterns
DMEM: Dulbecco’s modified eagle medium
ELISA: enzyme-linked immunosorbent assay
FBS: fetal bovine serum
HS: horse serum
LPS: lipopolysaccharide
NT: no treatment
P/S: penicillin/streptomycin
PAMPs: pathogen-associated molecular patterns
PFA: paraformaldehyde
PPRs: pattern recognition receptors
qPCR: quantitative polymerase chain reaction
RT: reverse transcription
RTKs: receptor tyrosine kinases
TLRs: Toll-like receptors
WT: wild-type.
