# Supplementary material for: Gas6 Inhibits Toll-Like Receptor-Mediated Inflammatory Pathways in Mouse Microglia via Axl and Mer
Source: Front Cell Neurosci. 2020 Oct 9;14:576650. doi: 10.3389/fncel.2020.576650 (PMC7584110; doi:10.3389/fncel.2020.576650)
Supplement: Supplementary file 1 [file Table_1.DOCX]

**Gilchrist *et al.* - Supplementary Material**

***
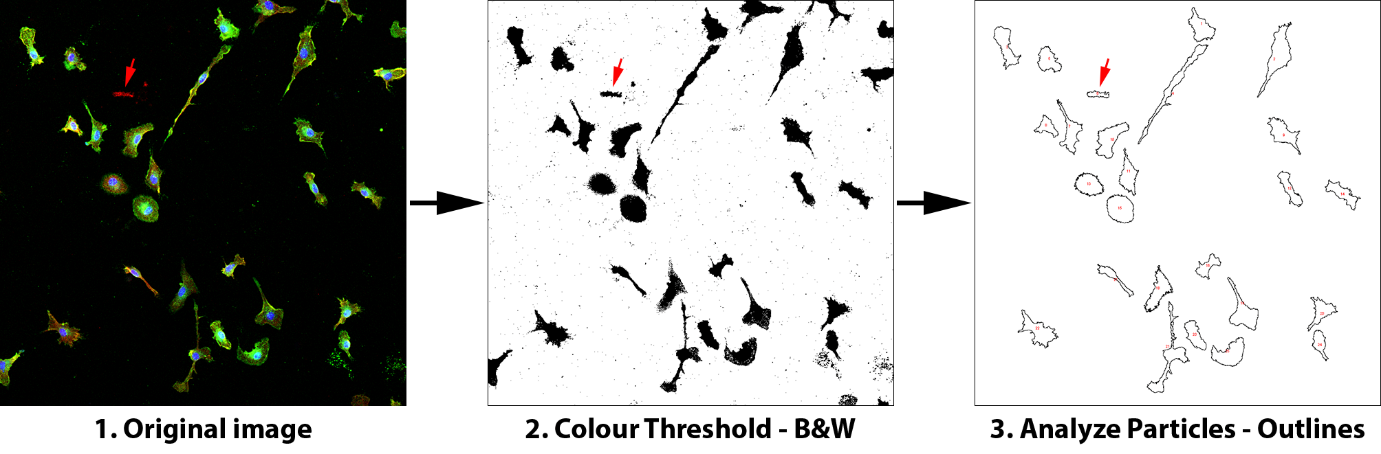
***

**Supplementary Figure S1. Workflow for image analysis of confocal images.** **(1🡪2)** Original images were converted to black and white (B&W) images using ‘Colour Threshold’ in ImageJ. **(2🡪3)** ‘Analyse particles’ created numbered outlines of all particles (i.e. cells) over 100μm and excluding those touching the perimeter. Large artefacts that weren’t clearly labelled with DAPI (blue), Iba1 (red) and β-actin (blue), illustrated by red arrows, were also excluded from analysis.

**
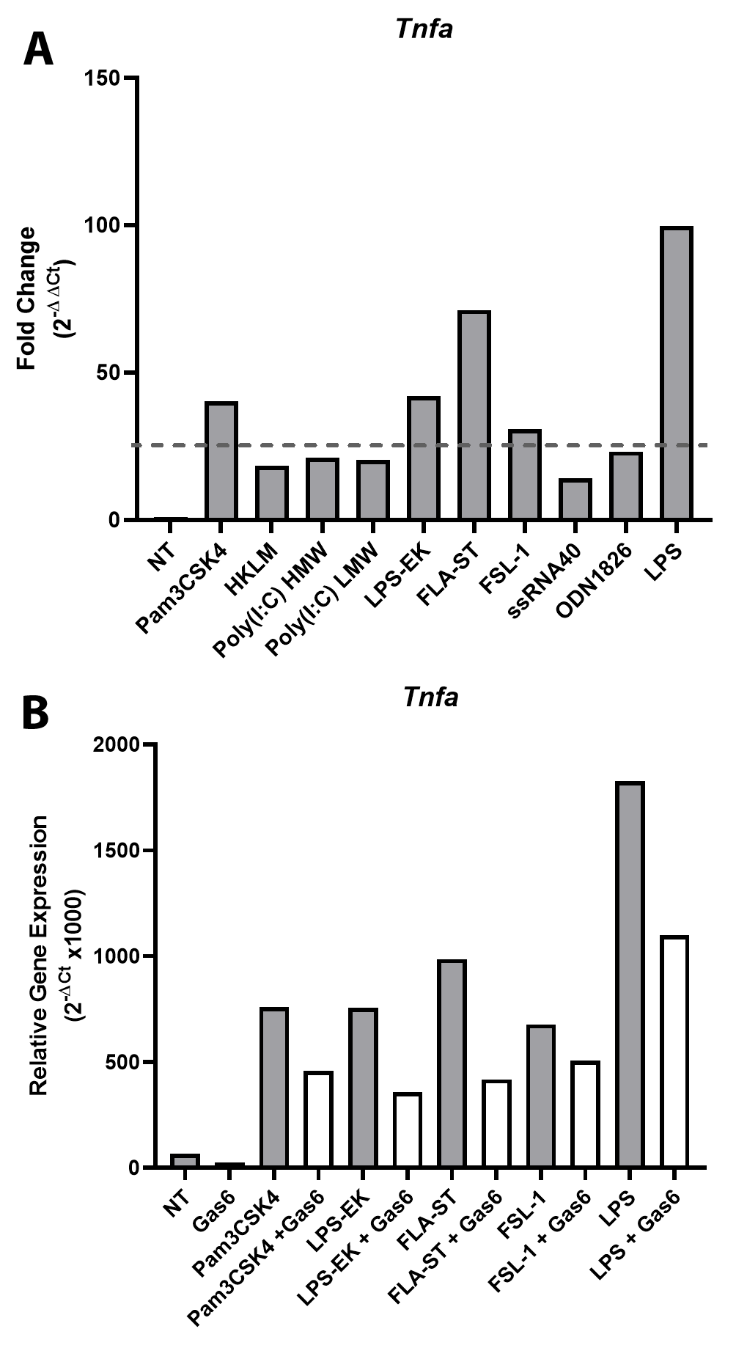
**

**Supplementary Figure S2. TLR screen for TNF-α in pure wild-type microglia cultures.** RT-qPCR was used to determine **(A)** fold-change of Tnfa gene expression with TLR agonist treatment relative to non-treated (NT) samples and **(B)** the relative gene expression with Gas6 pre-treatment for 1 hour before TLR agonist treatment. TLR agonists: Pam3CSK4 (0.1μg/mL) - TLR1/2 agonist; HKLM (10^7^ cells/mL) - TLR2 agonist; Poly(I:C) HMW (1μg/mL) - TLR3 agonist; Poly(I:C) LMW - TLR3 agonist (1μg/mL); LPS-EK (10ng/mL) - TLR4 agonist; FLA-ST (0.5μg/mL) - TLR5 agonist; FSL-1 (0.1μg/mL) – TLR 6/2 agonist; ssRNA40 (1μg/mL) – TLR7 agonist; ODN1826 (0.4μM) – TLR9 agonist; LPS (10ng/mL) – TLR4 agonist. TLR ligands that elicited a minimum 25-fold increase in Tnfa gene expression, as illustrated by the dashed line in **(A)** were selected for the Gas6 pre-treatment experiment. Graphs display data for one representative experiment.


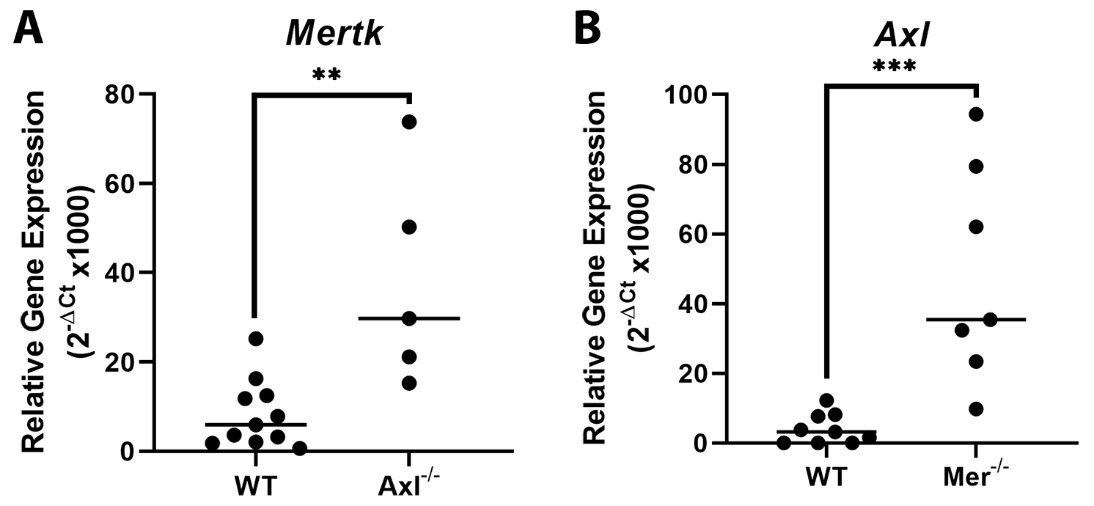


**Supplementary Figure S3. TAM single receptor knockout cells display higher expression of the remaining TAM receptor.** RT-qPCR was used to determine the relative gene expression (2^-ΔCt^) of Mertk in Axl^-/-^ microglia (A) or of Axl in Mer^-/-^ microglia (B). Gapdh was used as a reference gene. Data shown is individual data points with median bar for n=5-9 independent experimental repeats. Statistical significance was determined using Mann-Whitney test; **p<0.01; ***p<0.001.


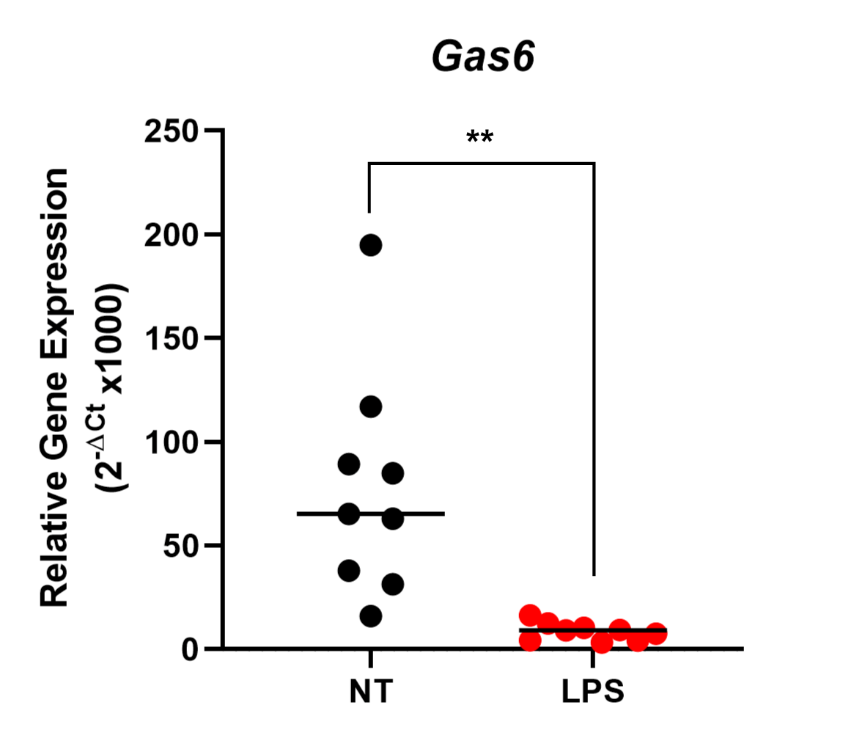


**Supplementary Figure S4. Expression of Gas6 in microglia is downregulated to negligible levels by LPS stimulation.** RT-qPCR was used to determine the relative gene expression (2^-ΔCt^) of Gas6 in wild-type microglial cells. Gapdh was used as a reference gene. Data shown is individual data points with median bar for n=9 independent experimental repeats. Statistical significance was determined using Wilcoxon signed-rank test; **p<0.01.

**
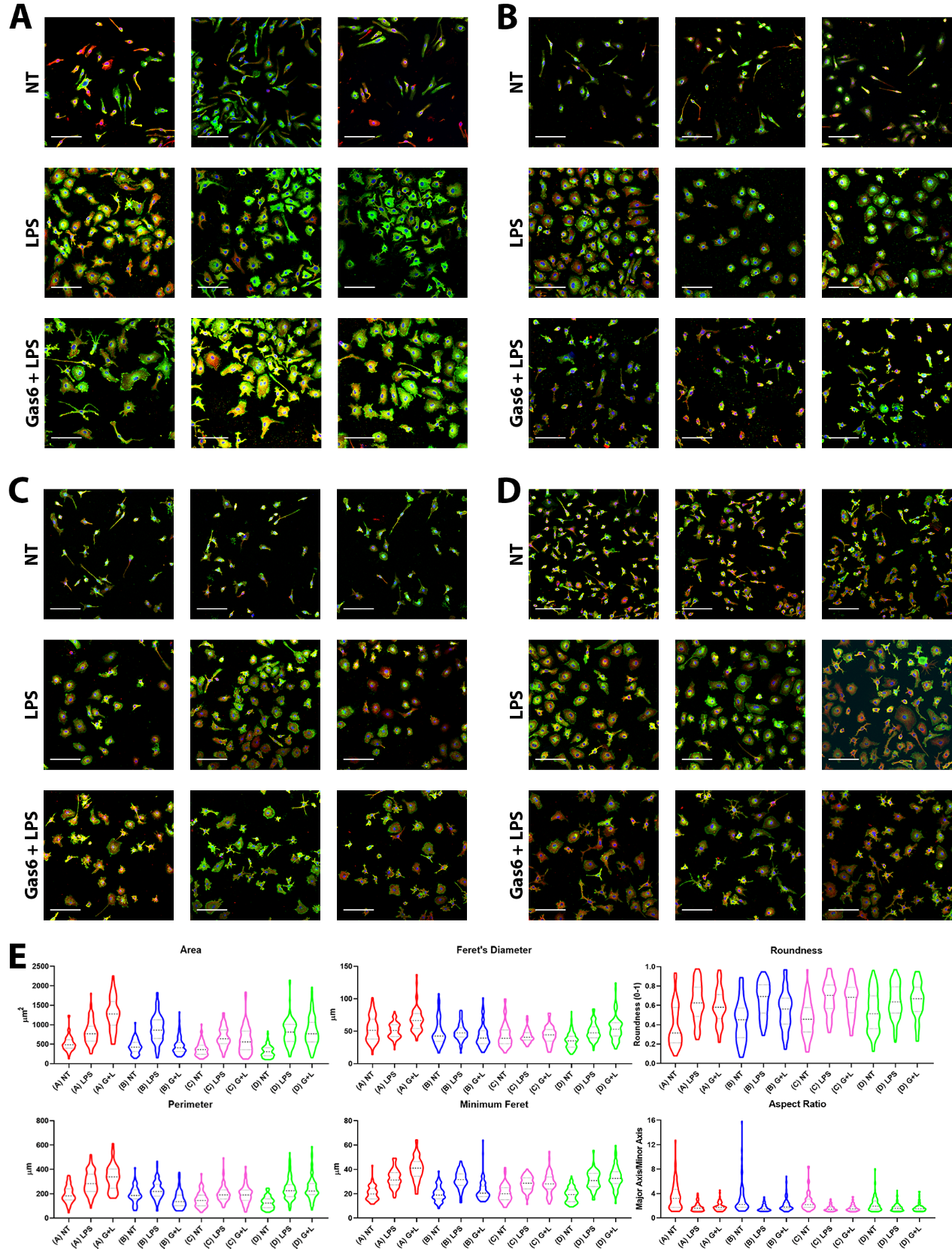
**

**Supplementary Figure S5. Individual images used to obtain morphological data.** **(A-D)** display four independent experimental repeats, each with no treatment (NT), LPS (10 ng/mL) for 19 hours and LPS with 1-hour pre-treatment with Gas6 (1.6μg/mL). Cells are stained with DAPI (blue), Iba1 (red) and β-actin (green). Scale bar = 100μm. **(E)** Quantification of morphological characteristics (area, Feret’s diameter, roundness, perimeter, minimum Feret and aspect ratio) for n=4 individual experimental repeats (A-D; n>300 cells per treatment group). Data is displayed as on violin plots showing median and upper/lower quartiles. NT – no treatment; LPS – 10ng/mL LPS (19 hours); G+L – 1.6μg/mL Gas6 (1 hour preceding LPS) + 10ng/mL LPS (19 hours).
